# Friction between human skin and incontinence pads in the presence of barrier protection products

**DOI:** 10.1177/09544119231178477

**Published:** 2023-06-10

**Authors:** Rachel Morecroft, Katherine Tomlinson, Roger Lewis, Matt Carré

**Affiliations:** Department of Mechanical Engineering, University of Sheffield, Sheffield, UK

**Keywords:** Friction, human skin, in vivo experiment, coefficient of friction, barrier treatments, incontinence, incontinence-associated dermatitis (IAD), moisture-associated dermatitis (MASD)

## Abstract

This novel experimental work aims to bring further knowledge of frictional performance of common barrier products used in the treatment of incontinence-associated dermatitis and determine how the skin-pad interface changes when a treatment is applied to the skin. Key data is reported and there is an in-depth analysis into friction profiles which reveals great differences between how different skin-pad tribosystems operate when exposed to commercially available barrier treatments. In a wet-pad state *Barrier cream A* (3M™ Cavilon™ Barrier cream) reduced friction and had much lower dynamic and static coefficients of friction than the other barrier treatments (*Barrier cream B* (Sorbaderm Barrier cream) and the *Barrier spray C* (Sorbaderm Barrier spray)). *Barrier cream A* provided stable friction coefficients in reciprocating sliding, whereas the other treatments, and untreated skin, did not display this unique characteristic. The barrier spray gave rise to high static friction coefficients and exhibited the most stick-slip. All three candidate barrier protection products were found to reduce directional differences in the static coefficient of friction: indicative of reduced shear loading. Knowledge of the desirable frictional properties would drive innovation in product development, and benefit companies, clinicians and users.

## Graphical abstract

**Graphical abstract:**
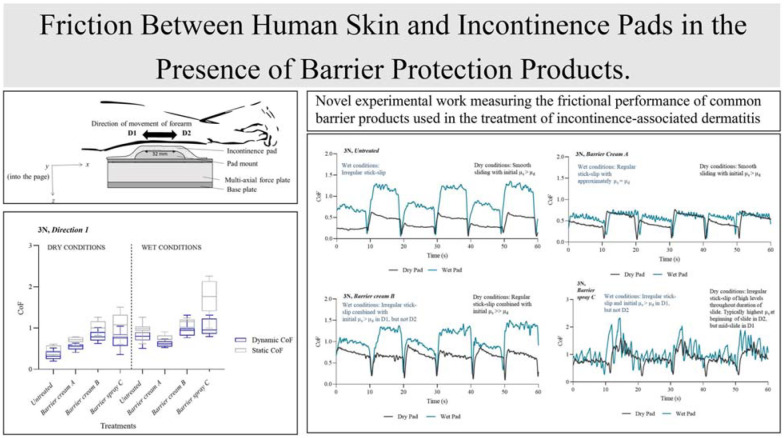

## Introduction

Skin irritation and discomfort can occur as a result of wearing incontinence pads, and although they are designed for maximum absorbency and comfort, the skin still maintains contact with urine and faecal matter, leaving the skin vulnerable to damage from moisture, friction, and bacteria. Barrier treatments are frequently used alongside incontinence pads to provide the skin with a protective waterproof barrier and to guard skin against rubbing, chafing, and irritation, along with promoting healing.^1^ Incontinence-associated dermatitis (IAD) is a form of moisture-associated dermatitis that develops when the skin has chronic exposure to urine and/or stool, and friction is also believed to have a significant role in IAD development.^2,3^ Sometimes friction is helpful, for example, grip on surfaces such as the soles of shoes, brake discs and in sport equipment such as yoga mats and rugby balls.^4^ However, when skin receives significant shear loading through frictional interactions, enough to damage the stratum corneum, then redness, inflammation, blistering, open wounds, and pressure sores can occur. Shear and friction are proven to pose a risk to skin health due to reducing blood flow to the area, eventually resulting in cell weakness, cell death, and tissue fragility.^5,6^ Skin damage can be managed by altering aspects of the tribosystem. The tribosystem involving an incontinence pad and skin is complex and may include the presence of excess wetness, urine, faeces, and different protective skin treatments. Different body shapes and sizes also complicate the research, and many different body areas can be affected by IAD; such as the buttocks, groin and upper thighs.^7^ Ageing skin may also present more complexity to the tribological properties due to the change in biophysical properties.^8^ There are a variety of absorbent products available for consumers to purchase, each with different absorbent capacitates, moisture wicking abilities, and material composition. Based on the culmination of these factors it is very likely that the tribosystem is unique for every individual who experiences IAD, which as a result means that people present with a variety of different symptoms to one another, as well as different severities.

There is a consensus amongst researchers that experimental parameters such as normal force, and sliding velocity influence the coefficient of friction (CoF). The main mechanism of skin friction is assumed to be adhesion^9,10^; however, it has become more widely recognised in recent years that deformation plays a great role.^7,11^ The area of the body tested also has an effect on the observed friction coefficient,^12^ due to the depth and deformability of the skin layers, surface topography,^13^ and the presence of subsurface anatomical features such as bony prominences. Skin moisture levels have also been shown to influence the coefficient of friction, with initial moisture absorption increasing the coefficient of friction.^14^ Being able to modify the skin-pad interface to the extent of lowering the dynamic and static CoF between the surfaces is assumed to present a scenario where the risk of incurring skin damage is lower. The skin-pad interface presents a hostile environment where skin is in a high moisture environment, making it a prime situation for skin to experience higher friction forces. A combination of hyper-hydration and chemical irritants from urine and faeces combined with mechanical factors such as friction and friction act together to weaken the structure of the stratum corneum and reduce the skin’s natural barrier defences.^15,16^

Skin treatments play an important role in helping to manage and prevent IAD, and often comprises a regime including cleansing, protecting and moisturising the skin.^17,18^ There are a wide range of products used to manage the condition, but very little research has been done to compare the frictional performance of treatments, which makes it difficult to understand the protective tribological mechanisms, benefits, and effects of treatments in the skin-pad interface. Knowledge of the desirable frictional properties would drive innovation in product development, and benefit companies, clinicians, and users. Some of the treatments are used to provide a protective barrier from moisture and friction, and others function as cleansers, moisturisers, or contain topical medicines like antibiotics, or antifungals. Determining the best ways to modify and optimise the skin-pad tribosystem will lead to improved management of skin health and could assist in the development of new treatments.

The aim of this study is to evaluate the friction interactions between the volar forearm surface and an incontinence pad in dry and wet conditions and test the effect of three topical treatments on the values of the dynamic coefficient of friction (DCoF) reported when the two surfaces are moving relative to one another, and static coefficient of friction (SCoF) reported on the initiation of sliding of the two surfaces.

## Materials and methods

### Test apparatus and method

A protocol was developed to assess tribological interactions in the skin-pad interface with IAD specific skin treatments and artificial urine present. Ethical approval was obtained from the Ethics Committee at The University of Sheffield (number 026173). Eight participants were recruited for the study (Table 3), with tests performed on the left volar forearm, which is a commonly used skin site for in vivo testing due to its ease of accessibility and being relatively hair free. The volar forearm is classed as thin skin, with four epidermal layers, this is the same as most areas of skin on the human body, except for palms of hands and soles of feet. Standard incontinence pads were used for testing, the incontinence pad unfolded was 215 mm long and 77 mm wide at its narrowest part, the flat surface of the pad stage was 32 mm long and 35.9 mm wide.

The incontinence pads were mounted on a multi-axial force plate (AMTI), which featured a HE6X6 force plate, a PJB-101 interface box and a PC, along with an RJ cable and a RS-232 cable. The principle by which the force plate works is based on the strain gauge flexibility technique, where three force components are measured in the x, y, and z-axes. The maximum normal force which can be tolerated in the z-axis is 44 N, but the device is also ideal for working with low loads. The experiments were conducted under dry and wet conditions. To achieve wet conditions 80 ml of artificial urine (a saline solution, composed of deionised water of 0.9 NaCl) was syringed onto the pad and left to absorb for 5 min before friction tests were conducted. Presented in this paper are results from tests conducted under a target 3 N normal load, alongside varying the skin treatments and wetness conditions in the interface.

**Figure 1.**
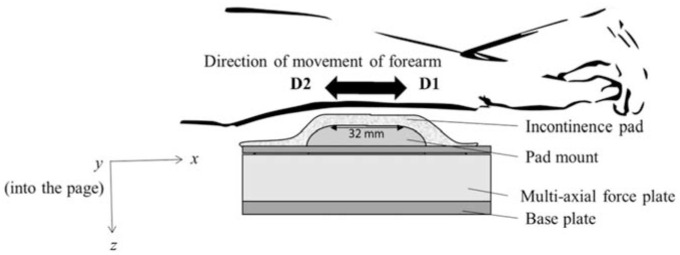
Friction rig and the volar forearm. Directions of the axes indicate that the x-axis is the general direction of movement of the forearm, the y-axis is the lateral movement, and the z-axis is the direction of the applied normal force.

In real-life scenarios, the applied load experienced on the skin exhibits significant variation across individuals. These variations can be attributed, in part, to the diverse locations where IAD can occur on the body, such as the buttocks, groin and inner thighs. A low normal load of 3 N was therefore selected to provide human participants comfort and control, allowing a repeatable test in which the different treatments could be compared. See Figure 1 for the experiment schematic.

The three treatments selected for the study were commercially available barrier creams and barrier spray, referred to as: *Barrier cream A*, *Barrier cream B* and *Barrier spray C*. The criteria for skin treatments to be included in the study were (i) commercial availability, (ii) clinical availability and/or being available on prescription, and (iii) being composed of different chemical constituent ingredients from one another. Barrier or emollient creams offer skin protection against bodily fluids and do not need to be applied too frequently as they are resistant to washing off. The differences between the active ingredients in *Barrier cream A* and *Barrier cream B* are shown in Table 1. Barrier or emollient sprays are fast drying and provide a barrier against bodily fluids, they are also marketed as offering protection against friction and can have the benefit of a non-contact application to irritated skin.

**Table 1.** Active ingredients in the barrier treatments.

Barrier treatment	Commercial name	Active ingredient
*Barrier cream A*	3M™ Cavilon™ Barrier cream	Dimethicone 1.3%, Acrylate Terpolymer, Diisooctyl Adipate, Coconut oil, Mineral oil, Glycerin/Glycerol, and Paraffin.
*Barrier cream B*	Sorbaderm Barrier cream	Ethylhexyl Isononanoate, Disiloxane, Acrylate Copolymer, Butylene Glycol, and Allantoin.
*Barrier spray C*	Sorbaderm Barrier spray	Hexamethyldisiloxane, isooctane, acrylate terpolymer, and polyphenylmethylsiloxane.

In total there were eight different test sessions for each participant, and these were conducted on separate days, separated by at least 48-h between each test session. All participants attended eight test days, where the test conditions were investigated, as shown in Table 2.

**Table 2.** Skin treatments and moisture conditions in the eight test conditions.

Test number	Skin treatment	Moisture condition
1	Untreated	Dry
2	Barrier cream A	Dry
3	Barrier cream B	Dry
4	Barrier spray C	Dry
5	Untreated	Wet
6	Barrier cream A	Wet
7	Barrier cream B	Wet
8	Barrier spray C	Wet

Prior to each experiment, participants’ skin was maintained in as close to a natural state as possible; they were instructed not to use any soap or other skin treatments on the forearm for at least 24 h prior to testing. During the preliminary session three skinfold calliper readings were taken from the test area of each participant to gain a quantitative indication of the thickness of the subcutaneous adipose tissue. The calliper reading, although a crude measure, was quick to conduct and gave an indication of the amount of adipose tissue in the forearm to therefore act as an indicator of the local compliance of the skin within the test area.^19^ The Corneometer^®^ CM825 probe (Courage-Khazaka, Cologne, Germany) was used to measure the hydration levels in the volar forearm up to a depth of 10–20 μm, capturing data solely from the superficial skin layers rather than tissue at greater depth.

The temperature of the volar forearm skin was measured and recorded prior to and after testing using an infrared thermometer. The volar forearm temperature recordings showed no significant changes in temperature from before and after tests in the dry conditions (*p* > 0.05). In the wet conditions all sites on average underwent cooling due to the room-temperature water that was applied to the pad.

Following this the test site was marked to indicate the sliding area and the treatment application area (15 × 7.5 cm) to which each product under test was applied. An amount of 0.2 ml of the barrier cream was applied to the treatment application area using a syringe and then the researcher used a nitrile gloved hand to distribute and rub in the treatment, resulting in a coverage of 1.78 × 10^−3^ ml/cm^2^. One spray of *Barrier spray C* was applied. The quantity of products applied was representative of consumer product application and the method was repeatable for testing. Treatments were left on the skin for 5 min before the friction tests were initiated, this gave a controlled window of time to prepare the participant for testing.

During the friction tests participants were able to observe and adjust the normal load by viewing the software interface as they positioned their arm onto the test rig. A 5 s timer was set to allow the participant to reach the desired 3 N load. A 10 s interval timer was then used to inform participants when to change the sliding direction and to achieve a target average sliding speed of 4 mm/s. The speed was chosen as it was considered to be both manageable and attainable by the participants, allowing for greater control of the movement of the forearm during the test. Figure 1 shows the experimental set up of the friction rig and volar forearm. Direction 1 (D1) is indicated in Figure 1, showing the movement of the hand back towards the force plate (and body) and direction 2 (D2) is indicated in Figure 1, showing the movement of the hand away from the force plate. The AMTI software was set to acquire 200 data points per second. Measurements were carried out between 20°C and 24°C, 30%–40% humidity.

### Participant information

For the eight participants further information and skin characterisation data are highlighted in Table 3.

**Table 3.** Participant information and skin characterisation.

Participant number	Age (years)	Sex	Moisture (c.u.) ± SD	Skinfold calliper (mm) ± SD
P1	21	F	24.9 ± 2.50	6.67 ± 2.31
P2	29	F	30.2 ± 2.98	4.67 ± 0.58
P3	30	F	34.2 ± 3.43	3.67 ± 0.58
P4	27	M	37.4 ± 1.63	2.00 ± 0.00
P5	26	M	48.0 ± 5.05	2.33 ± 0.58
P6	28	M	32.0 ± 1.35	4.67 ± 1.15
P7	27	M	52.9 ± 3.11	2.67 ± 0.58
P8	35	F	36.7 ± 2.36	10.67 ± 2.31

### Calculation of the Coefficient of Friction (CoF)

**Figure 2.**
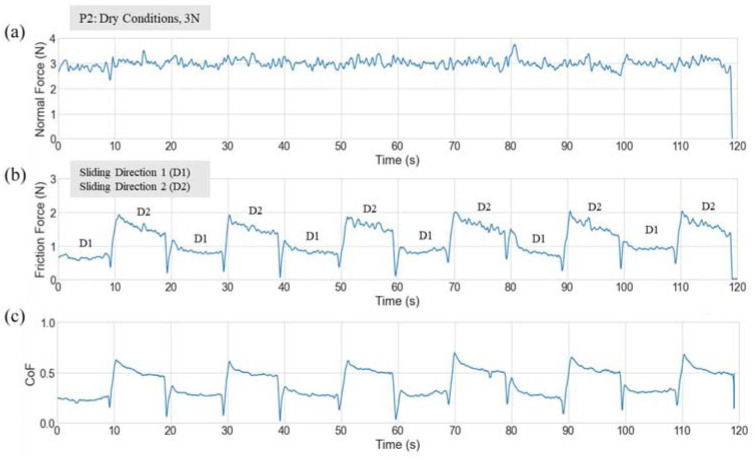
Normal force (*F_N_*), friction force (*F_R_*) and CoF during a dry-pad test. Part (a) is what the participant sees as part of the software interface allowing them to have a real-time view of normal force, part (b) shows the friction force of the full 12 slides as a resultant of the *F_x_* and *F_y_* components, and (c) is the calculated friction coefficient profile where *µ* = *F_R_*/*F_N_*.

**Figure 3.**
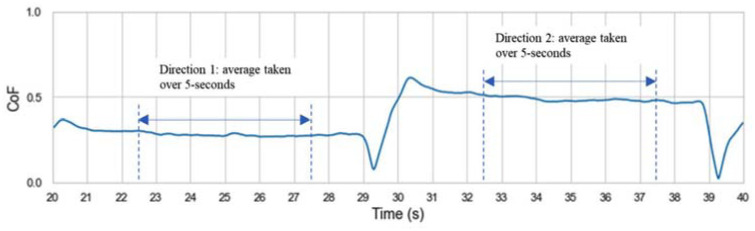
An extract of Figure 2 with one slide from D1 and one slide from D2. In each direction the average DCoF was calculated for a 5 s section within the mid portion each slide.

An example of force and friction profiles from a typical test run is shown in Figure 2, the data presented is from P2 in untreated dry conditions at a normal applied load of 3 N. The normal force profile for the full 120-s is displayed in Figure 2(a). The friction force Figure 2(b) was calculated using the resultant of the horizontal x and y friction components, where ‘Friction Force’ was the force acting in the plane normal to the applied load that resists the motion. The CoF profile determined from the raw data can be seen in Figure 2(c), where the y-axis is reported as CoF. Specific friction coefficient values required data extraction protocols which are described in Figure 3.

The direction of sliding directly impacted the magnitude of the CoF, see Figure 2(b) and (c). Therefore, it was necessary to calculate the (dynamic) DCoF separately for D1 and D2. It is expected that the higher shear force required on the reverse slide when tension has been applied to the skin results in the higher CoF. The method of extracting the DCoF is shown in Figure 3, where for each slide the average DCoF was calculated using data from the middle 5 s of each 10 s slide, which equated to 1000 data points. DCoF values for each test and sliding direction were then reported as an average of the 6 slides per direction, along with the corresponding standard deviations (SD).

### Friction profile analysis

**Figure 4.**
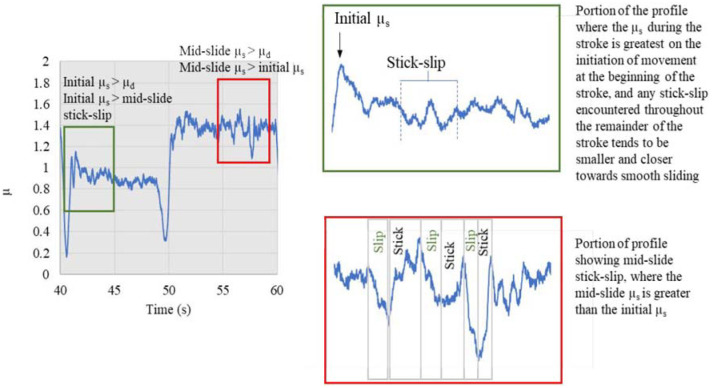
Stick-slip patterns in the friction profiles – Example curve is from P2 Barrier cream B, wet-pad conditions, 3 N normal force. (SCoF represented by $\mu_{s}$ , DCoF represented by $\mu_{d}$ ). First graph showing slide in both directions.

Studying the key features of friction profiles can greatly supplement the reporting of friction coefficients because they provide more insight into the reactions within the interface than a CoF alone can capture. This type of friction profile analysis is often missing in tribological work. This section presents a full set of CoF profiles for Participant 2 (P2), where the CoF has also been referred to as µ. A summary of some of the key friction characteristics that are present within a CoF profile are introduced in Figure 4. The static coefficient of friction (SCoF) is defined as the maximum CoF, at the point where sliding of the two surfaces is initiated.

The stick-slip action occurred when the pad was ‘stuck’ adhesively on certain areas of the skin causing the friction force to rise, until a slight separation happened between the two surfaces causing a rapid decline in the friction force. The slip action occurs when the shear force being applied is high enough to overcome the adhesion force. The experimental protocol was designed to measure the friction in two sliding directions however the frictional observations and values of the dynamic and static coefficient of friction are also representative of unidirectional sliding.

## Results

### CoF profiles for wet and dry pad conditions

**Figure 5.**
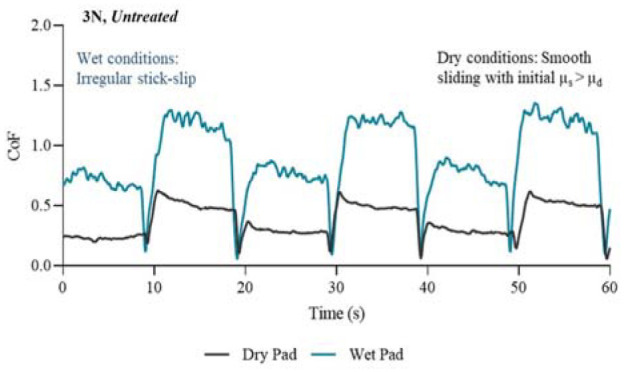
Friction coefficient profiles in wet and dry conditions for the first 60 s/6 slides of the experiment. Condition: 3N and untreated.

The presented profiles in Figure 5 to Figure 8 illustrate key features of the friction response associated with each treatment. The results are separated into four figures; each in either untreated or treated state, and at a normal force of 3 N. P2 was selected as an example because the CoF profile of this chosen participant consistently lay within the middle range of the other participants, therefore giving a good average representation of the CoF profiles for all participants.

**Figure 6.**
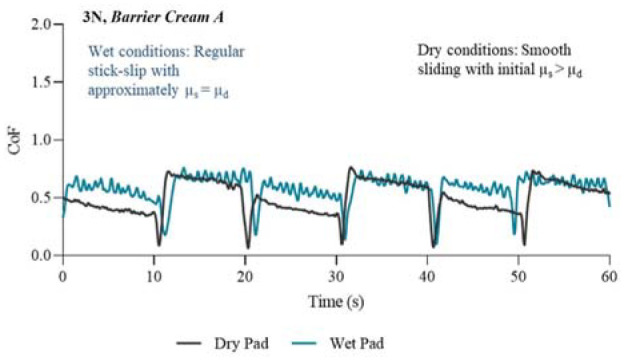
Friction coefficient profiles in wet and dry conditions for the first 60 s/6 slides of the experiment. Condition: 3N and treated with Barrier cream A.

**Figure 7.**
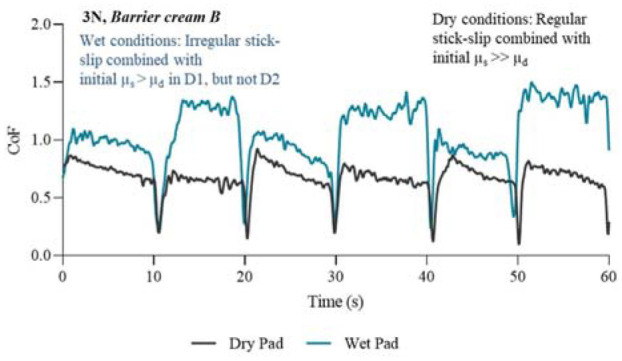
Friction coefficient profiles in wet and dry conditions for the first 60 s/6 slides of the experiment. Condition: 3N and treated with Barrier cream B.

**Figure 8.**
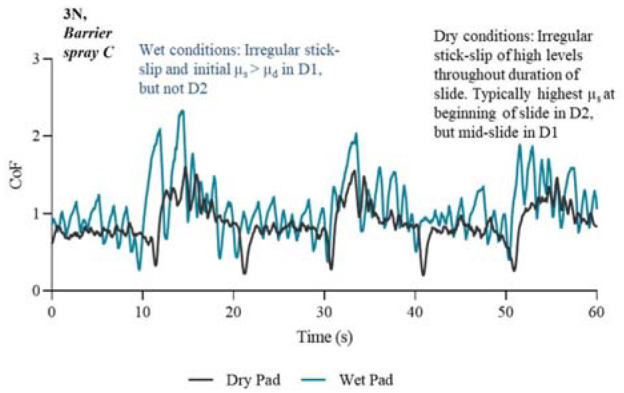
Friction coefficient profiles in wet and dry conditions for the first 60 s/6 slides of the experiment. Condition: 3N and treated with Barrier spray C.

Figure 5 shows the CoF profiles from the dry pad and wet pad in the first 60 s of the untreated skin friction test. The CoF in wet conditions was over a factor of two higher than in dry conditions. Over the first few seconds of the direction change the CoF increased, which happened until the friction between the two surfaces reached a peak (SCoF), and then sliding (DCoF) occurred. The wet conditions, as expected, had a higher CoF than the dry conditions, and the dry conditions had much less variation in amplitude as shown by the smooth profile. In previous research friction has been found to increase linearly with skin hydration.^7,20^ The wet pad condition had a greater amplitude during the dynamic portion of the CoF curve, which could be indicative of stick-slip interactions occurring between the pad and the skin, where the adhesive junctions between the surfaces become more difficult to overcome leading to a small increase in the friction coefficient.

Figure 6 displays the CoF profiles from the dry pad alongside the wet pad for the first 60 s of the friction test with *Barrier cream A* applied to the skin. Again, the dry profile is relatively smooth, while in the wet state stick-slip occurs again. However, the stick-slip is very ordered compared to untreated skin. This is termed ‘regular’ stick-slip, that is, the profile has a more consistent frequency and amplitude of fluctuation, compared to the other treated and untreated wet conditions. The stick-slip of *Barrier cream A* is characterised by uniform amplitude and frequency. *Barrier cream A* application may result in the surfaces being less likely to adhere for long periods which could be for several reasons, for example high hydrophobicity thereby repelling water off the skin and in doing so preventing changes to the structure of the stratum corneum. Alternatively, it could provide a hydrodynamic film to promote steady sliding compared to what the other treatments can achieve.

For *Barrier cream A* in wet conditions, D1 and D2 had similar friction coefficient values, a big contrast to the directional behaviour seen in untreated wet conditions. The shape of the profile suggests that the mechanism of effectiveness for *Barrier cream A* may be its ability to allow skin to slide over the pad with minimal to no tissue deformation. Another notable feature from Figure 6 is the overlapping profiles for wet and dry conditions, something which the *Barrier cream B* and untreated skin did not exhibit, which suggests that *Barrier cream A* works well to maintain lower levels of friction in an interface even when a dry pad becomes wet. *Barrier cream A* may have formed a lubricating layer by transforming the film into an emulsion thereby activating a hydrodynamic lubrication regime.

Figure 7 displays the CoF profiles from the dry pad and wet pad for the first 60 s of the friction test where *Barrier cream B* was applied to the skin. The key features of these profiles are stick-slip in both dry and wet conditions, and the duration of sticking is greater in wet conditions indicating that skin has been subjected to increased loading and associated shear strains and stresses. The level of CoF is higher with *Barrier cream B* than *Barrier cream A.*

In dry conditions the SCoF in D1 is higher than D2, see Figure 7, and the D1 DCoF starts off at a higher peak but reduced throughout the stroke to give a DCoF of similar value to D2. In wet conditions, the effects of the direction change on SCoF and DCoF are strong, giving rise to D2 with higher friction coefficients than D1. The type of stick-slip can be characterised as ‘irregular’ stick-slip. This result shows that when artificial urine interacts and combines with the *Barrier cream B* the friction increased to greater levels than skin containing no treatment. The wet conditions in the interface introduced increased tissue deformation and adhesion.

The barrier spray results showed that in both dry and wet conditions it increased the friction coefficients. The barrier spray CoF profile, shown in Figure 8, exhibited a very different shaped profile in the dry and wet conditions compared to the other two treatments.

Some directional effects in Figure 8 can be seen; a greater SCoF is reached in D2, and both sliding directions exhibit higher stick-slip amplitude and longer sticking time compared to the other treatments. All of the participants experienced irregular stick-slip as shown in Figure 8, although *Barrier Spray C* saw the largest spread of data of the tested treatments, as shown in Figure 10, indicating that the when the spray was applied there was a significant variation in the frictional performance based on individual participant characteristics. This observation may be attributed to the different biomechanical properties of the surface and subsurface tissue among the participants tested. Physically this manifested for the participants as a slight intermittent pulling sensation on the skin during sliding arm movement, which was often described as unpleasant. Sticking occurred more in wet conditions than dry conditions, where the large gaps between peaks represent the friction force building and then releasing. The amplitude of each peak reduced throughout any given D2 slide suggesting that when sliding is underway it becomes easier to break the adhesive junctions. As the tissue deforms during the slide it may also influence reducing the strength of the adhesion, meaning that the CoF falls throughout the stroke. During the change in direction, the barrier spray had sufficient time to form a strong bond between the skin and the pad so that the initial SCoF is high, followed by further stick-slips of decreasing ‘stickiness’ as the bonds become weaker during a slide. This scenario seemed to occur most when changing from D1 to D2, which is likely due to the loading set up by D1 pulling the skin in that direction first, causing a bias in that direction throughout the test. Subsequently, if skin experiences these loading conditions regularly, then over time it could become damaged.

Moisture in the contact would increase adhesion, and in all tested conditions both contact surfaces would have physically changed with the addition of the saline solution to the pad; resulting in the pad swelling as it became saturated, causing the top pad layer to become taut with a smoother surface. The increased moisture levels of the skin would also have resulted in a swelling and smoothing of the stratum corneum, and a loss of mechanical strength.^11^ With both surfaces becoming smoother and tauter the CoF increased due to the increase in contact area causing higher levels of adhesion and greater intermolecular attractions between the surfaces. It is also expected that when more moisture is absorbed by the skin, the skin becomes more elastic and adhesion and deformation increases. The static friction is higher than the dynamic friction because once the surfaces are moving relative to one another they have less time to adhere to one another.

The way water chemically interacts with the treatments in the interface will be key to their frictional performance and the shape of their friction coefficient profiles. A treatment may stay in place, form an emulsion, transfer to the pad, or cause the skin surface to change either causing a higher or lower real contact area. Overall, the application of *Barrier cream A* resulted in adhesive junctions being broken more quickly and uniformly. In contrast, an irregular stick-slip is suggestive of greater adhesion and deformation leading to temporary sticking and subsequent CoF growth.

### Friction coefficients

The mean results and standard deviations, across the eight participants, for all treatments in wet and dry tests in both direction 1 (D1) and direction 2 (D2) are presented in Table 4. In wet conditions *Barrier cream A* is the only treatment to produce a lower CoF than untreated skin. *Barrier spray C* has the highest CoF in all situations. The CoF is higher in direction 2 in both wet and dry conditions for each treatment.

**Table 4.** Static (SCoF)and Dynamic (DCoF) Coefficient of Friction data for all treatments in wet and dry conditions in direction 1 (D1) and direction 2 (D2).

	Untreated	*Barrier cream A*	*Barrier cream B*	*Barrier spray C*
SCoF (±SD) Dry D1	0.46 (±0.11)	0.71 (±0.07)	1.03 (±0.15)	1.06 (±0.31)
SCoF (±SD) Wet D1	0.98 (±0.18)	0.75 (±0.10)	1.13 (±0.10)	1.75 (±0.44)
SCoF (±SD) Dry D2	0.67 (±0.14)	0.73 (±0.08)	1.05 (±0.18)	1.28 (±0.34)
SCoF (±SD) Wet D2	1.30 (±0.17)	0.84 (±0.15)	1.38 (±0.18)	2.24 (±0.37)
DCoF (±SD) Dry D1	0.34 (±0.11)	0.55 (±0.08)	0.80 (±0.13)	0.73 (±0.25)
DCoF (±SD) Wet D1	0.79 (±0.14)	0.62 (±0.07)	0.95 (±0.17)	1.02 (±0.20)
DCoF (±SD) Dry D2	0.53 (±0.12)	0.60 (±0.09)	0.86 (±0.13)	0.89 (±0.25)
DCoF (±SD) Wet D2	1.16 (±0.14)	0.69 (±0.11)	1.17 (±0.16)	1.27 (±0.21)

**Figure 9.**
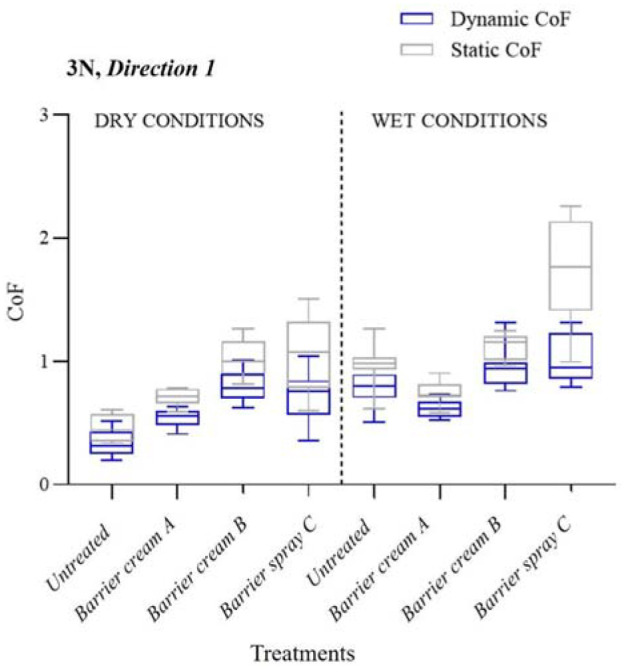
SCoF and DCoF according to different treatments applied in wet and dry conditions. The results are for 3 N in Direction 1.

**Figure 10.**
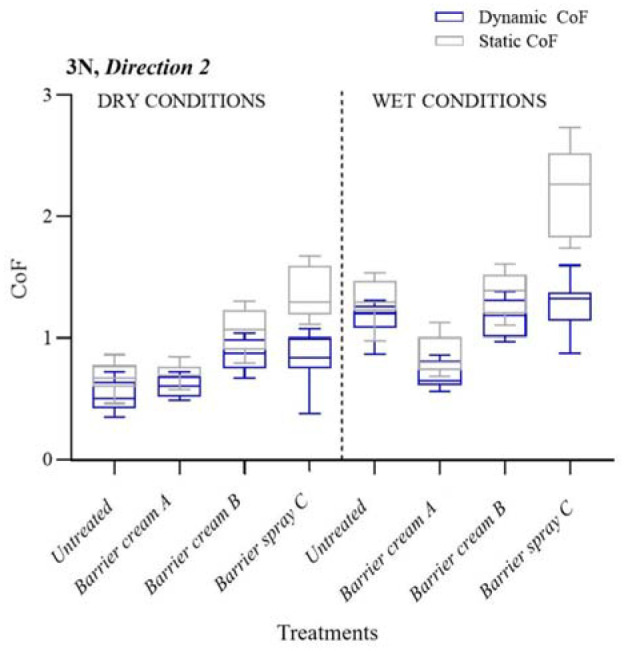
SCoF and DCoF according to different treatments applied in wet and dry conditions. The results are for 3 N in Direction 2.

The dynamic and static friction coefficients in dry and wet conditions at 3 N are shown in Figure 9 (D1) and Figure 10 (D2). The interquartile range is shown in the boxplot with the median represented by the horizontal line and the whiskers extending to the minimum and maximum values.

The boxplot overlay in Figure 9 for D1 shows that each treatment in both wet and dry conditions had different sized intervals between DCoF and SCoF, so the way the tribosystem interacts in each system differs at the start of the stroke and mid-stroke depending on the treatment applied. As expected, in all states the SCoF was statistically significantly higher than that of the DCoF (*p* < 0.05), though *Barrier cream B* and *Barrier spray C* both showed a greater difference between the two values, highlighting that before sliding occurs the skin undergoes a greater friction force, which is indicative of greater shear occurring.

The DCoF of untreated wet skin was over twice as high as the value for dry skin, demonstrating, as expected, that wet conditions significantly increased friction in the skin-pad interface. In other experiments in literature, it was found that CoF increased after application of water to the skin, and slowly over a period of 20–30 min of drying time the skin hydration returned to the original levels.^21–23^

Amongst all treatments, *Barrier cream A* had the lowest DCoF, SCoF and interquartile range compared to other treatments, indicating that it would be more suitable to minimise friction when compared to the other treatments tested in this work. *Barrier cream A* was also able to provide the most consistent skin friction conditions independent of the participant it was applied to. The SCoF was on average highest for skin treated with a spray in wet conditions, to varying degrees depending on the participant, as shown by the large interquartile range in Figure 9. The high SCoF of the spray is potentially a cause for concern in maintaining skin integrity; the repeated high friction and shear on the skin could weaken the SC and reduce healthy blood flow to the underlying tissue in the case of excessive prolonged shear and cyclic deformations.

Notable features exhibited by barrier spray friction tests were difficulty of movement, high SCoF, and both recorded and visible stick-slip which became more extreme in wet conditions. The change in direction (D1 and D2) had an influence on the value of the DCoF and SCoF recorded, and in general, D2 values were higher than those reported in D1, Figure 10. The directional change in friction coefficients may be due to a ‘bow-wave’ formation^11^ whereby skin is compressed due to the horizontal motion of the skin across the pad, and stretched in the other. Kwiatkowska et al.^11^ also found this to be accompanied by substantial lateral skin deformation. The other deformation occurs in the deeper layers of the skin due to the application of normal force on the skin from the contacting pad.

## Discussion

*Barrier cream A, Barrier cream B* and *Barrier spray C* are marketed as having many of the same benefits, such as being ‘wash-off resistant’, ‘not blocking absorbency of incontinence pads’, and ‘providing a waterproof barrier to protect against bodily fluids and high friction’. Despite these common benefits, in this work it has been found that tribologically they perform very differently. With *Barrier cream A* being the only treatment to reduce friction in the wet-pad interface, its application as a friction modifier to lower friction within the skin-pad interface is supported by the findings of this study. The application of *Barrier Spray C* produced irregular stick-slip, and resulted in a tacky sensation on the skin during the arm motion. This effect could be due to an ingredient called acrylate terpolymer which helps create a film. The dimethicone in the spray could potentially have a plasticising effect on the skin as it is commonly used as a moisturising agent.

The low CoF with *Barrier cream A* applied is most likely a result of a reduction in both the adhesion and deformation components of the friction. *Barrier cream A* may primarily function as an interfacial lubricant by reducing micro stick-slip. The variability of the friction behaviour of different treatments has also been discussed in literature; according to Holroyd and Graham^23^‘the evidence does suggest there is variability in the efficacy and the ability of commercial products to protect the skin, prevent maceration, and maintain adequate skin health’. They also point out that it is ‘essential to carry out an individualised assessment on each patient to ensure the optimum management plan is in place’. Masen et al. found in a comprehensive study of the lubricative performance of a variety of creams and skin treatments that coconut oil and beeswax offered long-lasting low friction.^24^*Barrier cream A* contains coconut oil which may be one of the key components within the treatment that provided the unique friction characteristics that were observed in this study.

Potentially the presence of *Barrier cream A* in a tribological interface can enable adhesive connections to be broken more uniformly, and/or it may have reduced the strength of the smaller and weaker molecular attractions between the surfaces for example, Van der Waals forces. Notably, *Barrier cream A* also was the only treatment in this work to decrease the DCoF and SCoF in wet conditions, meaning it was the best all round treatment for maintaining low levels of friction in a skin-pad interface. In everyday practice, individuals are likely to apply the cream just once before undergoing several dry-wet cycles. If friction levels could be maintained at a constant level throughout these cycles, then this would likely offer the most protection from the development of IAD, in that case *Barrier cream A* appears to offer this consistency.

Another factor to consider with the application of the barrier creams is they are designed to remain on the skin for long periods of time, so interaction with a pad surface should theoretically not result in treatment being removed from the skin. All treatments tested in this experimental work incorporate polymer ingredients into their formula which contributes to the long-lasting nature of the product on the skin, upwards of 24-h. However, in a situation with high friction forces in the interface there is increased likelihood of sloughing treatment from the skin and transfer to the pad. Destruction of the integrity of the treatment layer could negatively impact the protective shield that the treatment provides to the skin. The friction altering behaviours of the treatments could also have been achieved due to treatments changing the contact area, impacting the height of asperities, initiating the formation of hydrodynamic films and/or by altering molecular attractions between the surfaces.

The treatment identified in this study with the most versatility in protecting skin is *Barrier cream A* because it reduced DCoF and SCoF in wet conditions compared to the untreated wet skin, whereas none of the other treatments had this effect. Additionally, in dry conditions the application of *Barrier cream A* did not significantly increase the DCoF or SCoF compared to the untreated skin in dry conditions. Again, both other skin treatments did not share this protective friction response. However, skin treated with any of the three barrier treatments was found to reduce the percentage difference between D1 and D2 SCoF showing that by applying a skin treatment then deformation or ploughing of the skin during sliding was minimised.

## Conclusions

The volar forearm skin-pad experimental protocol developed in this research was designed to be representative of loading conditions experienced by pad users living with incontinence-associated dermatitis. Friction profiles recorded during the different treatments and conditions are illustrated and discussed in detail, which provides great insight into the types of frictions interactions and mechanisms. The friction at the interface was shown to be affected by the sliding direction, the hydration of the incontinence pad and the barrier treatment used.

The sliding direction was shown to affect the CoF, resulting in a higher value on the reverse slide. The motion in the reverse direction directly follows tension being applied to the skin in the opposing direction, requiring a higher shear force to overcome the adhesive force at the interface. This motion could be compared to moving from sitting to standing or movement whilst sitting or lying down and is highly relevant to the consideration of the incontinence pad-skin interface.

In wet conditions both the SCoF and the DCoF were higher than dry conditions for all candidate treatments, however, the level of this increase was much less compared to untreated skin. In dry conditions the barrier treatments all produced a higher level of friction than untreated skin. In wet conditions *Barrier cream A* (3M™ Cavilon™ Barrier cream) was the only treatment to reduce both static and dynamic friction. *Barrier cream B* (Sorbaderm Barrier cream) and *Barrier spray C* (Sorbaderm Barrier spray) increased friction in all situations compared to untreated skin, with *Barrier spray C* increasing friction the most. Barrier sprays are beneficial for non-contact applications and for providing a fast-drying waterproof layer, however, *Barrier spray C* (Sorbaderm Barrier spray) was found to produce a high level of friction in both wet and dry conditions.

These findings combined, point towards *Barrier cream A* (3M™ Cavilon™ Barrier cream) being the best treatment to prevent higher friction coefficients, as well as minimising surface and subsurface shear. Additionally, a predictable skin response like 3M™ Cavilon™ Barrier cream produces is ideal in terms treating medical conditions because a prescriber or advisor can be confident about the skin response for most people.
